# Baseline Troponin T level in stroke and its association with stress cardiomyopathy

**DOI:** 10.1371/journal.pone.0209764

**Published:** 2018-12-31

**Authors:** Kai Liesirova, Eugenio Abela, Thomas Pilgrim, Laura Bickel, Thomas Meinel, Julia Meisterernst, Verma Rajeev, Hakan Sarikaya, Mirjam R. Heldner, Tomas Dobrocky, Erick Siqueira, Marwan El-Koussy, Urs Fischer, Jan Gralla, Marcel Arnold, Heinrich P. Mattle, Kety Hsieh, Simon Jung

**Affiliations:** 1 Department of Neurology, Inselspital, Bern University Hospital, University of Bern, Bern, Switzerland; 2 Department of Cardiology, Inselspital, Bern University Hospital, University of Bern, Bern, Switzerland; 3 University Institute for Diagnostic and Interventional Neuroradiology, Inselspital, Bern, Switzerland; Azienda Ospedaliero Universitaria Careggi, ITALY

## Abstract

**Background:**

Differential diagnosis of elevated high sensitive Troponin T (hsTnT) in acute ischemic stroke includes myocardial infarction (MI) and neurogenic stunned myocardium (NSM). The aim of this study was to identify factors associated with baseline hsTnT levels and MI or NSM in acute ischemic stroke.

**Methods:**

We studied 204 consecutive patients of the prospective acquired Bern Stroke Database with acute ischemic stroke diagnosed by brain MR. All patient histories and cardiac examinations were reviewed retrospectively. Volumetry of lesions on diffusion and perfusion weighted brain imaging (circular singular value decomposition, Tmax >6sec) was performed. Voxel based analysis was performed to identify brain areas associated with hsTnT elevation. Linear regression analysis was used to identify predictors of baseline hsTnT levels and myocardial infarction.

**Results:**

Elevated hsTnT was observed in 58 of the 204 patients (28.4%). The mean age was 68.3 years in the normal hsTnT group and 69.7 years in the elevated hsTnT group. Creatinine (p<0.001, OR 6.735, 95% CI 58.734–107.423), baseline NIHSS score (p = 0.029, OR 2.207, 95% CI 0.675–12.096), ST segment depression (p = 0.025, OR 2.259, 95% CI 2.419–35.838), and negative T waves in baseline ECG (p = 0.002, OR 3.209, 95% CI 13.007–54.564) were associated with hsTnT elevation, while infarct location and size were not. Coronary angiography was performed in 30 of the 204 patients (14.7%) and myocardial infarction was diagnosed in 7 of them (23.3%). Predictive factors for myocardial infarction could not be identified.

**Conclusion:**

Elevated baseline baseline hsTnT was associated with NIHSS, creatinine, ST segment depression and inverted T waves, but not with stroke location or size. None of the factors was helpful to differentiate MI and NSM. Therefore, ancillary investigations such as coronary angiography, cardiac MRI or both may be needed to solve the differential diagnosis.

## Introduction

About 18–20% of ischemic stroke patients show elevated high sensitive Troponin T (hsTnT) levels on admission [1–3]. Differential diagnosis includes renal failure, chronic heart failure, myocardial infarction (MI) and stress-related cardiomyopathies or so called neurogenic stunned myocardium (NSM). NSM, which is characterized by contraction band necrosis on the cellular level, can clinically be expressed by hsTnT elevation, electrocardiography (ECG) abnormalities (such as ST segment changes, T inversions and pathological Q waves), regional wall motion abnormalities and in its most extend as Takotsubo Cardiomyopathy. In some patients diagnostic differentiation between MI and NSM is straightforward using ECG and echocardiography, but in some patients differentiation is impossible due to overlapping diagnostic criteria (ST segment changes, T inversions and pathological Q waves, wall motion abnormalities).[4–7]

A common practice in many stroke centers is to consider slightly elevated hsTnT levels originating from the brain and highly elevated levels from the heart. However, this empirical assumption might not be accurate, because MI patients have an increased risk for stroke not only during the acute stage but also later on, when the cardiac wall lesions enhance the risk for thrombus formation.[8] Accordingly, stroke may occur after MI when enzyme levels are already decreasing or even normalized. Indeed, MI can be found in stroke patients with only marginally elevated hsTnT.[9] Therefore, MI may be underdiagnosed in patients with acute ischemic stroke and the question arises whether baseline hsTnT levels have any predictive value to differentiate NSM and MI.

Studies showing an association between brain areas that are thought to be involved in the pathogenesis of NSM in stroke and baseline hsTnT elevation suggest that baseline hsTnT and stroke location might potentially differentiate between and MI, but results so far have been conflicting.[10–14] In a case control study, Ay and coworkers found the right posterior, superior, and medial insula and the right inferior parietal lobe to be associated with hsTnT elevation.[10] Unlike Ay et al, Laowattana and coworkers reported that left insular stroke was associated with an increased risk of adverse cardiac outcome and decreased cardiac wall motion compared to stroke in other locations and TIA.[15] Sykora et al demonstrated that baroreflex impairment in acute stroke is associated with insular involvement, left and right.[16] Beside the parts of the limbic system the rostral ventrolateral medulla [17], solitary tract nucleus, and intermediolateral cell column are thought to be brain areas that are potentially prone to produce NSM.[5]

The aim of this study was to identify baseline characteristics, laboratory parameters, ECG findings and imaging parameters (size and location of stroke) that are associated with baseline hsTnT levels and MI in a large cohort of acute ischemic stroke patients and to address the clinical question whether baseline hsTnT levels are helpful to differentiate between NMN and MI.

## Patients and methods

### Demographic data and review of cardiac history and examinations

This study is based on the Bernese stroke registry, a prospectively collected database of all patients with acute stroke admitted to our stroke center. Some aspects have been reported previously.[18–21]

All acute stroke patients between 2011 and 2014 were included in the analysis if 1) stroke was diagnosed by MRI on admission 2) and if hsTnT and ECG were performed on admission in the emergency department.

Age, gender, medication, baseline National Institutes of Health Stroke Scale (NIHSS), time from symptom onset to treatment, atrial fibrillation, hypertension, diabetes, smoking, hypercholesterolemia, history of ischemic stroke, known prior myocardial infarction, known coronary heart disease, prior medication, stroke treatment details such as intravenous thrombolysis or endovascular treatment and complications were recorded prospectively as baseline characteristics.

Laboratory changes during hospitalization, ECG findings and echocardiographic studies, and—if performed—coronary angiographies were systematically reviewed (K.L., T.P.) in all included patients. Myocardial infarction was suspected in case of a characteristic rise and fall of creatinine kinase MB fraction or hsTnT in combination with at least one of the following: ischemic symptoms, new pathologic Q-waves, or ischemic electrocardiographic changes. In addition, MI was only suspected if an underlying higher grade coronary stenosis or culprit lesion was found in coronary angiography.

The study was approved by the local ethics committee of Bern. Data were anonymized after data collection.

### MRI methods and image analysis

Pre-treatment MRI was performed using a 1.5T or 3T MR imaging system (Magnetom, Siemens). The MRI protocol included whole brain DWI (b = 1000t, 24 slices, thickness 5 mm, TR 3200ms, TE 87ms, number of averages 2, matrix 256×256) yielding isotropic b0 and b1000 as well as apparent diffusion coefficient (ADC) maps that were calculated automatically. Volumetry of lesions on diffusion and perfusion weighted imaging (circular singular value decomposition, Tmax >6sec) was performed with the FDA approved software Olea Sphere (Olea Medical) (K.H., M.E.K.).

Voxel-based lesion-symptom mapping was used to identify neuroanatomical regions associated with elevated hsTnT levels. Binary lesion masks were generated from the DWI data by manual segmentation using MRIcroN imaging software (https://www.nitrc.org/projects/mricron/). These masks were then normalized according to localization using standard Montreal Neurological Institute (MNI) space with SPM12 (http://www.fil.ion.ucl.ac.uk/spm/).

### Assessed parameters

The following parameters were assessed (from database or by retrospective reviewing of patient history): age, gender, baseline NIHSS score, blood pressure on admission, blood markers on admission (hsTnT, CK, CK-MB, Creatinine), arterial hypertension, atrial fibrillation, prior myocardial infarction, coronary heart disease, prior medication (antiplatelet agents, anticoagulants, antihypertensive drugs, betablockers, statins), hypercholesterolemia, diabetes, smoking, prior stroke or TIA, diffusion restricted volume, hypoperfusion volume, perfusion-to-diffusion mismatch ratio, ECG changes on admission (ST-elevation/depression, T-negativity, pathological Q-wave, left/right bundle branch block, incomplete right bundle branch block, left anterior fascicular block) and echocardiographic abnormalities (wall motion abnormalities, left atrial and left ventricular thrombi). hsTnT was measured with ElektroChemiLumiszenImmunoAssay (ECLIA) from Roche (cutoff ≤0.014 μg/l).

### Statistical analysis

Statistical analysis was performed using SPSS 21 (SPSS Inc., Chicago, Illinois, USA). Bivariate analysis of categorical variables was performed with χ2 and Fisher’s exact test as appropriate and continuous variables with Mann-Whitney test. Non-normally distributed variables were transformed with the best method achieving near normal distribution (rang transformation for hsTnT, log transformation for Creatinine, and square root transformation for NIHSS). Linear regression with inclusion of all parameters which showed at least a trend for an association (p<0.2) was used to determine the predictors of hsTnT elevation and MI. A p-value < 0.05 was considered significant.

Association between baseline hsTnT and lesion load at each voxel was tested with two mass-univariate general-linear models: first, by an independent-samples t-test between patients with elevated and non-elevated hsTnT, and second, by regression of hsTnT against lesion load. Both models included total lesion volume as a nuisance variable. Only voxels lesioned in at least 2 subjects were included. Significance was tested using permutation tests in NiiStat (https://www.nitrc.org/projects/niistat/), i.e. by deriving an empirical distribution of T-values across all voxels and testing the probability of the original value against this distribution. We used 4000 permutations and considered a voxel statistically significant if its value survived a False-Discovery Rate (FDR) corrected threshold of p<0.05.

## Results

A total of 204 consecutive patients with ischemic stroke were included in this study. Elevated hsTnT was observed in 58 of the 204 patients (28.4%). Mean hsTnT levels were 0.063 μg/l (SD 0.628) in all patients and 0.209 μg/l SD 1.172) in those with elevated hsTnT above the 99^th^ percentile (> 0.014 μg/l). Transthoracic echocardiography was performed in 41 patients and transesophageal echocardiography in 95 patients.

Baseline characteristics and the results of univariable analysis for association with baseline hsTnT are shown in Table 1. All variables with p<0.2 were entered in linear regression analysis. Linear regression analysis revealed Creatinine (p<0.001, OR 6.735, 95% CI 58.734–107.423), NIHSS (p = 0.029, OR 2.207, 95% CI 0.675–12.096), ST segment depression (p = 0.025, OR 2.259, 95% CI 2.419–35.838), and negative T waves in baseline ECG (p = 0.002, OR 3.209, 95% CI 13.007–54.564) to be independently associated with hsTnT elevation (Table 2). The DWI lesion volume remained in the model until NIHSS was added to the analysis. When atrial fibrillation was included in the model, it turned out as independent predictor (p = 0.015, OR 2.475, 95% CI 4.977–45.574) and ST segment depression dropped out. The strength of this latter model was weaker, probably due to a significant dropout of patients (atrial fibrillation status was documented only in 100 of the 204 patients).

**Table 1:** Baseline characteristics of 204 acute stroke patients. P values of univariable analysis association with baseline hsTnT as continuous variable.

	Patients without hsTnT elevation (n = 146)	Patients with hsTnT elevation (n = 58)	*p*
Age	68.3±13.5	69.73±13.1	0.537
Male gender	94/146 (64.4%)	41/58 (70.7%)	0.924
NIHSS base score	7.1±6.3	10.4±9.5	<0.001
Atrial fibrillation in long-term ECG monitoring	27/67 (40.3%)	18/33 (54.5%)	0.051
Prior MI	5/86 (5.8%)	7/30 (23.3%)	<0.001
Coronary artery disease	13/146 (8.9%)	17/58 (29.3%)	<0.001
Prior stroke	6/89 (6.7%)	5/37 (13.5%)	0.130
Diabetes	22/145 (15.2%)	15/58 (25.9%)	0.006
Hypertension	98/146 (67.1%)	45/58 (77.6%)	0.040
Hypercholesterolaemia	84/146 (57.5%)	34/58 (58.6%)	0.672
Smoking	33/144 (22.9%)	9/57 (15.8%)	0.978
Antiplatelets/oral anticoagulation use	62/146 (42.5%)	38/58 (65.5%)	<0.001
Statin drug use	35/146 (24.0%)	22/56 (39.3%)	0.018
Antihypertensive drug use	67/146 (45.9%)	40/57 (70.2%)	<0.001
Betablocker use	39/146 (26.7%)	19/58 (32.8%)	0.036
BP systolic	162.7±29.7	166.1±31.3	0.340
BP diastolic	88.0±19.0	85.8±25.7	0.660
**Laboratory parameters**			
Creatinine baseline	80.1±17.7	106.0±42.1	<0.001
**ECG Parameters**			
ECG ST-depression	35/146 (24.0%)	18/57 (31.6%)	0.072
ECG ST-elevation	10/146 (6.8%)	5/57 (8.8%)	0.079
ECG T-negative wave	16/146 (11.0%)	12/57 (21.1%)	0.009
ECG pathological Q-wave	25/146 (17.1%)	17/57 (29.8%)	0.224
ECG Left bundle branch block	2/146 (1.4%)	2/57 (3.5%)	0.179
ECG Right bundle branch block	6/146 (4.1%)	3/57 (5.3%)	0.342
ECG incomplete right bundle branch block	1/146 (0.7%)	0/57 (0.0%)	-
ECG left anterior fascicular block	5/146 (3.4%)	3/57 (5.3%)	-
**Echocardiography parameters**			
Ventricular wall motion abnormalities	16/105 (15.2%)	11/27 (40.7%)	0.006
Left atrial thrombus	3/108 (2.8%)	0	1.0
Left ventricular thrombus	1/106 (0.9%)	0	1.0
**MRI Parameters**			
MRI diffusion restricted volume in ml	13.6±27.8	14.9±19.1	0.358
MRI perfusion restricted volume in ml	50.1±57.3	66.9±74.5	0.193
MRI perfusion/diffusion mismatch ratio	10.2±15.1	5.7±7.6	0.910

**Table 2:** Independent predictors of baseline hsTnT elevation.

	OR	p	95% CI
**Dependent variable: baseline hsTnT**			
Creatinine	6.735	<0.001	58.734–107.423
NIHSS	2.207	0.029	0.675–12.096
ECG ST-depression	2.259	0.025	2.419–35.838
ECG negative T wave	3.209	0.002	13.007–54.564

Coronary angiography was performed in 30 of the 204 patients (14.7%). After reviewing all available data MI was diagnosed definitely in 7 of the 30 patients (23.3%). Predictive factors for MI could not be identified.

**Fig 1:**
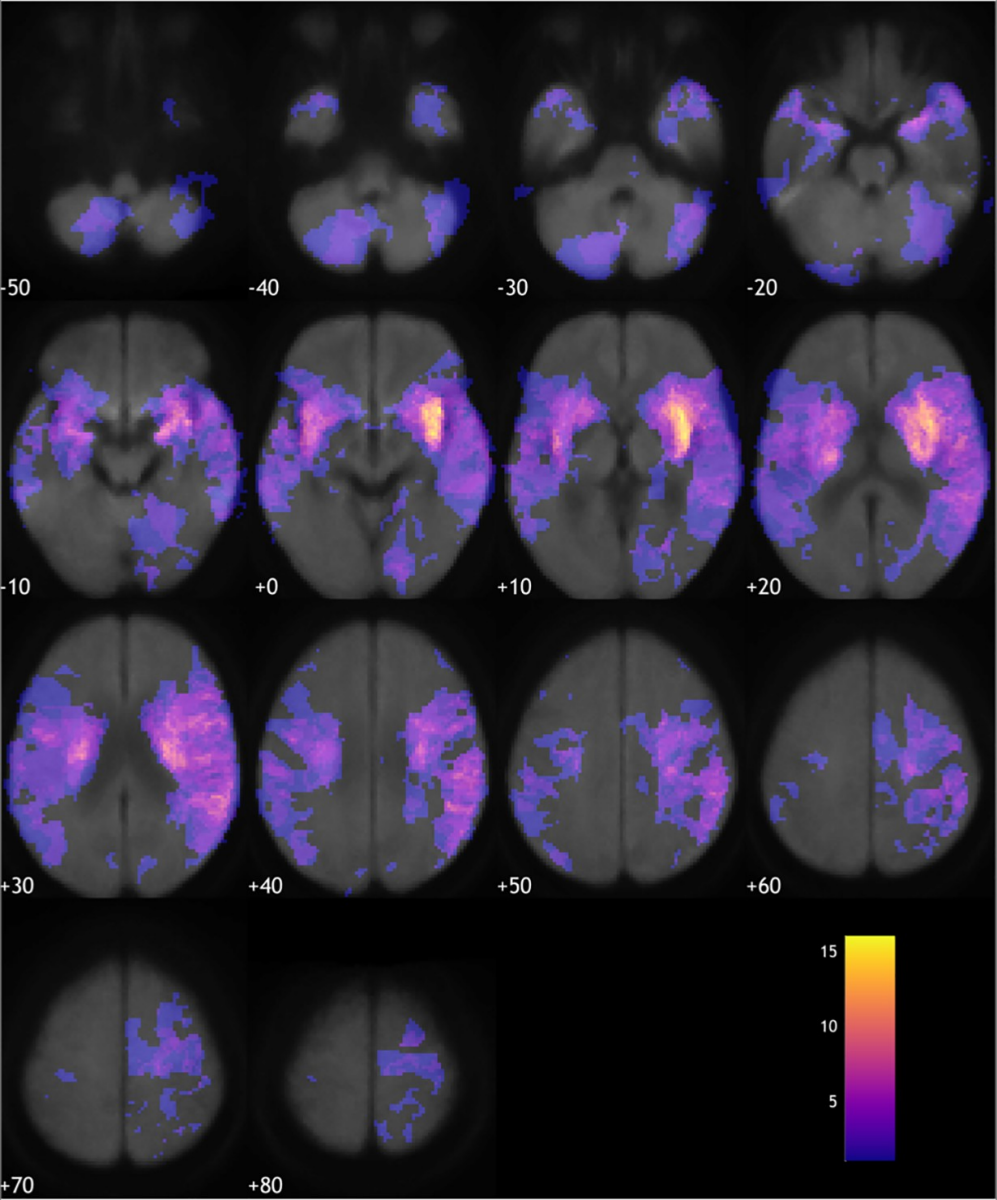
Lesion overlap map. This figure shows a color coded map indicating the number of subjects that have a lesion at a particular voxel, overlaid on an average diffusion-weighted image in standard Montreal Neurological Institute space (left in image is left in the brain, coordinates indicate position along the z-axis, i.e. inferior to superior).

MRI quality was sufficient for voxel-based analysis in 149 patients. A lesion overlap map is shown in Fig 1. Voxel-based lesion-symptom mapping failed to reveal unique lesion-hsTnT associations. In neither the categorical comparison of patients with and without elevated hsTnT nor the direct regression between hsTnT-values and lesion masks did any voxel survive the prespecified statistical threshold. Subgroup analysis with exclusion of patients with elevated creatinin failed to reveal unique lesion-hsTnT associations as well.

## Discussion

The main finding of our study is that baseline hsTnT is probably not a suitable marker to differentiate MI and NSM in patients with acute ischemic stroke. The NIHSS score was associated with baseline hsTnT, which may suggest some impact of NSM on hsTnT. Nevertheless, we did not identify any predictors of MI that help to differentiate between MI or NSM as potential reasons of hsTnT elevation. hsTnT elevation was associated with ST segment depression and negative T waves in ECG, both of which can result of MI or NSM.

Overall, 28.4% of stroke patients included in this analysis had elevated hsTnT at baseline. In accordance with previous studies, we identified renal failure to be associated with hsTnT elevation.[13,22] In addition, ST segment depression and negative T waves on baseline ECG were associated with hsTnT elevation. As both MI and NSM can be accompanied by ST segment depression and negative T waves, these ECG findings do not help to differentiate between the two etiologies.[4,5] In accordance with previous studies hsTnT elevation and stroke severity as measured by NIHSS scores were also associated [11,12,23,24]. However, in our study unlike in previous investigations, neither the size of diffusion restriction nor the size of perfusion deficit in Tmax maps, was associated with hsTnT.[6] Because NIHSS scores are influenced both by infarct size and location, it might be that infarct location is more important for NSM development than infarct size. Indeed, several brain areas have been shown to be involved in the development of NSM, especially the insular cortex but also some brain stem areas. Nevertheless, whether baseline hsTnT elevation is associated with any infarct location remains uncertain, because study results are conflicting: three studies described involvement of the right insular cortex and right inferior parietal lobe to be associated with baseline hsTnT elevation,[10–12] but other studies did not.[13,14] In our detailed voxel based analysis we did not find any association of infarct location with baseline hsTnT. Interestingly, a recent study found an association of infarct location with temporal changes of hsTnT but not with baseline hsTnT.[14]

Only seven of our 30 patients who underwent coronary angiography were diagnosed to have suffered from MI, indicating that a high proportion of hsTnT elevation is based on NSM, renal failure or other concomitant diseases. Nevertheless, because coronary angiography was performed in only 15% of our patients and no reliable markers to distinguish MI from NSM were identified, MI was probably missed in a relevant number of patients who did not undergo coronary angiography.

Our study has several limitations. It is a retrospective observational study with probable selection bias and a relative small sample size. In particular, the decision to perform coronary angiography was made individually, and therefore the rate of coronary heart disease in those patients who did not undergo coronary angiography cannot be reliably estimated. Furthermore, some parameters were not available of all patients, which may have reduced the statistical significance.

To summarize, we identified an association of NIHSS and baseline hsTnT but not between specific brain areas and hsTnT when affected. This indicates that NSM may occur with high NIHSS scores. However, our analysis did not identify any factor that was specific for NSM or MI. None of the factors associated with elevated hsTnT turned out to be helpful to differentiate NSM and MI in the clinical setting. Accordingly, MI and NSM have to be taken into account in all patients with elevated baseline hsTnT levels. To solve the differential diagnosis ancillary investigations such as cardiovascular MR or coronary angiography or both may be required. A prospective study with systematic differentiation of MI and NSM using cardiovascular MR or coronary angiography in patients with elevated hsTnT might improve our understanding of the significance of elevated baseline hsTnT and its temporal changes.

## Supporting information

S1 Dataset Minimaldataset. (SAV)
